# Resource-sharing in multiple-component working memory

**DOI:** 10.3758/s13421-016-0626-7

**Published:** 2016-06-10

**Authors:** Jason M. Doherty, Robert H. Logie

**Affiliations:** Human Cognitive Neuroscience, Department of Psychology, University of Edinburgh, 7 George Square, Edinburgh, UK EH8 9JZ

**Keywords:** Working memory, Attention, Memory

## Abstract

Working memory research often focuses on measuring the capacity of the system and how it relates to other cognitive abilities. However, research into the structure of working memory is less concerned with an overall capacity measure but rather with the intricacies of underlying components and their contribution to different tasks. A number of models of working memory structure have been proposed, each with different assumptions and predictions, but none of which adequately accounts for the full range of data in the working memory literature. We report 2 experiments that investigated the effects of load manipulations on dual-task verbal temporary memory and spatial processing. Crucially, we manipulated cognitive load around the measured memory span of each individual participant. We report a clear effect of increasing memory load on processing accuracy, but only when memory load is increased above each participant’s measured memory span. However, increasing processing load did not affect memory performance. We argue that immediate verbal memory may rely both on a temporary phonological store and on activated traces in long-term memory, with the latter deployed to support memory performance for supraspan lists and when a high memory load is coupled with a processing task. We propose that future research should tailor the load manipulations to the capacities of individual participants and suggest that contrasts between models of working memory may be more apparent than real.

Working memory refers to the support of online cognition involving both processing and temporary memory. Some theories of working memory assume a single, domain-general, attention-based resource, the capacity of which varies between individuals. This approach has been very successful in exploring what drives the correlation between individual differences in measures of working memory capacity and individual differences in a wide range of other cognitive abilities (e.g., Daneman & Hannon, 2007; Kane & Engle, 2002, 2003; Unsworth, Brewer, & Spillers, 2009; Unsworth & Engle, 2007). However, this approach has been rather less focused on the functional organization of the cognition that supports measured working memory capacity. Here, there has been a debate as to whether working memory might comprise a general-purpose attentional resource that combines temporary activation of long-term memory with the current focus of attention (e.g., Cowan, 2005; Cowan, Rouder, Blume, & Saults, 2012), time-limited switching and allocation of attention (e.g., Barrouillet et al, 2004; Barrouillet & Camos, 2001, 2007, 2010), or might comprise multiple specialized resources in working memory that can act in concert with one another and with activated long-term memory to support task performance (e.g., Baddeley, 1986, 2012; Baddeley & Hitch, 1974; Baddeley & Logie, 1999; Logie, 1995, 2011; Logie & Niven, 2012). The latter has been referred to as the multiple-component model (Baddeley & Logie, 1999). Here we present two experiments investigating the relationship between storage and processing during dual tasking—specifically, whether participants’ performance on a memory or processing task is affected by the load of the concurrent task.

The multiple-component model refers to the coordinated deployment of multiple cognitive resources, each of which has its own resource limitations serving a specific function in online cognition. One component, the phonological loop, has been proposed for temporary storage of a sequence of phonological codes in serial order (e.g., Baddeley, 1992). A second component, the visual cache, is thought to store an array of visual items or a single visual item that may vary in complexity (Logie, 1995, 2003, 2011). In the original version of the model (Baddeley, 1986; Baddeley & Hitch, 1974), a central executive was thought to be a central processing and control mechanism. Subsequently (Baddeley, 1996; Baddeley, Emslie, Kolodny, & Duncan, 1998; Logie, 2016), there has been reference to a range of different executive functions, associated respectively with inhibition, updating, task switching (Miyake et al., 2000), dual-task coordination (Logie, Cocchini, Della Sala, & Baddeley, 2004), retrieval from long-term memory (Unsworth & Engle, 2007), and the generation and manipulation of mental images (Borst, Niven, & Logie, 2012; van der Meulen, Logie, & Della Sala, 2009).

The basis of the multiple-component model is that of separate, specialized resources, and so it predicts little effect of concurrent task demand on memory performance when each component is operating within its own capacity limits, but a general dual-task cost when one or more components is at or beyond its own capacity limit (e.g., Duff & Logie, 1999, 2001; Logie & Duff, 2007). Further evidence that temporary memory does not necessarily draw on processing capacity in working memory has come from a series of studies (e.g., Anderson, Bucks, Bayliss, & Della Sala, 2011; Baddeley, Logie, Bressi, Della Sala, & Spinnler, 1986; Baddeley, Bressi, Della Sala, Logie, & Spinnler, 1991; Cocchini, Logie, Della Sala, MacPherson, & Baddeley, 2002; Logie et al., 2004; MacPherson, Della Sala, Logie, & Wilcock, 2007; Salthouse, Fristoe, Lineweaver, & Coon, 1995) demonstrating that when healthy participants are asked to perform two distinct tasks concurrently (such as oral serial ordered recall of aurally presented digit sequences together with a perceptuomotor tracking task), then performance of each task is very little different from when performing only digit recall or performing only perceptuomotor tracking. This accumulated evidence suggests further that the processes thought to support oral serial recall, such as subvocal rehearsal, can operate even when a demanding gestural-motor task is being performed at the same time. However, this is only true if the digit recall task involves recall of sequences set at the digit span for each participant and at the maximum tracking speed at which each individual participant can follow the moving target. If storage and processing demands are set at levels above the storage or processing capacities of an individual, then there is evidence of a performance cost relative to performing processing alone, or memory alone (e.g., Logie et al., 2004). However, Logie et al. (2004) also demonstrated that when a dual task cost was observed, the extent of this cost did not vary as a function of the varying cognitive load of each task. A similar result was reported by Baddeley and Hitch (1974) when they combined maintenance of a six-letter list with an interpolated reasoning task that varied in difficulty. There was an overall cost to time for reasoning of the concurrent memory load, but no effect on memory performance and no difference in the dual task cost for the more difficult compared with the easier reasoning problems. The set of results from these studies contrasts sharply with the assumptions of a single, general-purpose attentional mechanism.

The time-based resource-sharing (TBRS) model is one example of a theoretical framework for working memory that assumes a single, limited capacity, general-purpose, attention-based system that constrains online processing of information as well as the maintenance of temporary memory traces (e.g., Barrouillet et al., 2004; Barrouillet et al., 2007; Barrouillet & Camos, 2001, 2007, 2010). The TBRS model states that the rate of short-term forgetting that participants display relies on the attentional demand (or “cognitive load”) of concurrent tasks, because memory traces are assumed to decay when they are not reactivated or “refreshed” by attention.

Barrouillet and colleagues have demonstrated a cognitive load effect in a number of studies in the years since the publication of the original outline of their model (for a recent review, see Barrouillet & Camos, 2015). We argue that because the procedure for working memory experiments often require participants to complete tasks set at a high difficulty level (perhaps beyond the capacity of individual working memory components), this may lead to an emergent shared-resource effect and potentially blur any separation of working memory components. For example, in Experiment 2 from Barrouillet et al. (2004), participants were required to complete continuous reading and operation span tasks (i.e., reading lists of equations, or reading lists of equations and calculating and providing the solutions) while simultaneously memorizing strings of consonants. Barrouillet et al. reported that the higher cognitive load of the operation span task compared to the continuous reading span task and an articulatory suppression condition resulted in poorer memory performance. They interpreted this as suggesting that the high cognitive load prevented rehearsal of the memory set. However, it is important to note that Barrouillet et al. (2004) describe how a number of trials were not included in the analysis of operation span data because some participants failed to answer, answered incorrectly, or faltered during their responses. The authors note that such interruptions were not present in the reading span or articulatory suppression conditions, suggesting these conditions were considerably easier for participants.

Being the seminal study investigating the TBRS theory of working memory, Barrouillet et al.’s (2004) paper has influenced the methodology of the TBRS literature, including the possibility of “overloading” working memory components due to an insensitivity to participants’ individual ability. A number of subsequent TBRS papers explicitly describe the exclusion of participants or individual trials from analyses due to high error rates (e.g., Barrouillet et al., 2007; Camos, Lagner, & Barrouillet, 2009; Vergauwe, Barrouillet, & Camos, 2009, 2010), suggesting that the task demands were beyond the capacity of working memory resources of some participants. The tasks reported in the TBRS studies may therefore provide a good measure of maximum operating capacity of working memory when the overall task demand exceed the overall working memory capacity. At these very high cognitive loads, data patterns could give the impression of performance being constrained by a single, limited capacity system while being insensitive to the contribution to that maximum operating capacity of discrete, specialized components.

Researchers interested in individual differences in mental capacity are concerned with the overall capacity limit of the system and so the arguments above do not pose a problem for them. However, Logie (2011) noted that the multiple-component framework for working memory is less concerned with this emergent overall capacity, and rather more concerned with the underlying cognitive mechanisms that contribute to this overall capacity. For example, individual differences in working memory capacity could be explained by both the capacity of components working in concert to support overall performance levels, and by the assumption that additional components are recruited to provide support for task performance when overall task difficulty increases beyond the capacity of individual components.

One more recent approach within the TBRS literature assumes two separate maintenance mechanisms (rehearsal and attentional refreshing) investigated by Camos et al. (2009). The authors reported the disruptive effects of cognitive load and of articulatory suppression are additive, suggesting separate involvement of both rehearsal and attentional refreshing in the maintenance of verbal memory items. Likewise, Vergauwe, Camos, and Barrouillet (2014) reported an effect of increasing memory load on processing reaction time (an effect that is considerably larger under articulatory suppression), suggesting separable mechanisms of rehearsal and refreshing for maintenance of verbal memory items. The proposal for rehearsal is compatible with the proposal of an articulatory (Baddeley, 1986) or phonological (Baddeley, 1992) verbal rehearsal loop. The proposal for refreshing is consistent with the contribution of activated and reactivated long-term memory traces to support temporary retention in working memory in addition to rehearsal (e.g., Borst et al., 2012; Logie, 1995; Unsworth & Engle, 2007). However, the conditions under which both rehearsal and refreshing might be used compared with only rehearsal or only refreshing remain unclear.

Unsworth and Engle (2006, 2007) proposed a framework relevant to the question of above-span performance and the possible role of nonphonological processes in verbal memory storage. In a review of simple and complex span literature, Unsworth and Engle (2007) found that simple span (memory without ongoing processing) predicted higher order cognitive abilities to the same extent as complex span (memory in the context of ongoing processing) as long as performance was measured using long lists: the magnitude of the correlations between fluid intelligence and performance on simple span and complex span converge on a similar value at longer list lengths for the simple-span task. Reviewing data from multiple papers, the authors note that simple span performance is more reliant on phonological processes and so is more disrupted by articulatory suppression, phonological similarity among items in the list, and by lists of longer compared with shorter words. Unsworth and Engle note that complex span most likely relies on phonological processes in addition to the processes that correlate with higher order cognition. They propose that simple span performance on shorter lists depends on primary memory (PM), and that as list lengths increase, items are displaced and stored in secondary memory (SM). In complex span tasks, memory items are displaced from PM by the concurrent processing task and are retained in SM. The correlation between long-list simple span tasks and fluid intelligence, and between complex span tasks and fluid intelligence, is therefore proposed to be driven by individual ability in retrieving items from SM. It is likely that the recruitment of these nonphonological processes for recall of longer lists accounts for predictive power of simple span tasks that use longer lists. It is also interesting to note the implication that complex span tasks, also referred to as working memory span tasks, might be measuring ease of retrieval from secondary memory, or long-term memory, and therefore might not actually be measuring the capacity of working memory. In sum, Unsworth and Engle’s results suggest that when demands on memory in simple span exceed the capacity of temporary verbal memory, then other resources in the cognitive system contribute to overall performance. This has the implication that with high memory loads, memory performance may be driven by retrieval from a verbal representation in long-term memory that might be disrupted by attentional refreshing, whereas low memory loads may rely on a phonological representation in temporary verbal memory that is disrupted by articulatory suppression.

The experiments described in this article measured verbal memory and spatial processing performance in single- and dual-task conditions, with the aim of investigating the effect of manipulating task demand based on participants’ individually measured single-task simple span. A dual-task combination of verbal memory storage and spatial processing was chosen to allow interpretation of any effects in terms of attentional trade-off rather than due to domain-specific interference that might arise from combining verbal memory with verbal processing. Based on Baddeley’s (1992; Camos et al., 2009; Logie, Della Sala, Laiacona, Chalmers, & Wynn, 1996) concept of a phonological loop, we assume a domain-specific verbal short-term memory component of working memory. Once the capacity of the phonological loop is reached participants can support memory performance by drawing on other cognitive resources. Whether this memory support involves increased reliance on strategies (Logie et al., 1996), the use of visual codes (e.g. Logie, Saito, Morita, Varma, & Norris, 2015; Saito, Logie, Morita, & Law, 2008), storage and retrieval of items stored in secondary memory (Unsworth & Engle, 2006, 2007)—or some other process, such as “refreshing”—we would argue that such a process requires attention above and beyond what is required for at-span or below-span lists, that would rely on temporary storage and rehearsal in the phonological loop. Therefore, increasing memory load beyond a participant’s span under dual-task conditions should have a measurable effect on concurrent processing performance as additional maintenance mechanisms are employed to support memory. It is also possible that participants recruit multiple resources for memory performance at span in single-task conditions, and that the introduction of a concurrent processing task taxes this same combination of resources. For example, Saito et al. (2008) demonstrated that participants may use both visual codes and phonological codes to retain visually presented verbal material. We would still expect an effect of memory load on processing performance that is more pronounced for longer lists due to task demand exceeding the capacity of the resources used to support span, requiring an increased involvement of domain-general attentional resources.

Conversely, due to the specialized nature of the verbal memory component of working memory, we would not expect that increasing the demand of the processing task beyond a participant’s capacity would have an impact on at-span memory performance: domain-general resources involved in the processing task can support above-span memory performance, but the rehearsal mechanisms of the verbal memory store can do nothing to support above-span processing performance.

## Experiment 1

### Method

#### Participants, stimuli, and apparatus

Twenty-four undergraduate students took part for course credit (seven male and 17 female, mean age = 20.8 years, range = 18–27 years). Data were collected using 36-cm × 27-cm displays set at 800 × 600 pixel resolution and 60 Hz refresh rate situated approximately 50 cm from participants with keyboards to record responses.

#### Procedure

Each participant completed six conditions in total: single-task memory and processing conditions, and four combined memory and processing dual-task conditions. The single-task conditions were counterbalanced yet always preceded the dual-task conditions. The dual-task conditions were fully counterbalanced, resulting in 24 counterbalanced conditions.

Each participant’s memory ability was measured using a span procedure in which he or she was required to recall number sequences of increasing length. Numbers appeared in the center of the screen consecutively for 1,000 ms each and were followed by a 6,000-ms fixation cross (+). Following this retention interval, participants were asked to enter the numbers in order into an onscreen text box using the numeric row on the keyboard. Each level (e.g., four items to remember, five items to remember) featured five randomly generated lists, and participants were required to accurately recall at least 4/5 lists to move on to the next level. This continued until participants were unable to reach this criterion, at which point their memory span was calculated as the average length of the last five correctly recalled lists. For example, if a participant correctly recalled 4/5 five-item lists, and 1/5 six-item lists, their memory span would be calculated as 5.2. This 4/5 criterion was chosen as a sensible cutoff at which participants begin to perform below ceiling and so would provide an accurate sensitive measure of memory capacity, and also to provide some parity with the processing task described below. The strictness of the criterion also ensured that the difficulty of the processing task would be set at an appropriate level for the introduction of a concurrent processing task.

Participants’ spatial processing ability was also measured using a span procedure in which they were instructed to judge whether successive “boxes” were situated in the top or bottom half of the screen, and answer via a key press. The location of boxes deviated randomly within a +-15 to +-20 pixel range vertically, and +-150 pixels horizontally (though no two consecutive processing stimuli could appear within 130 pixels of each other). This equates to an average vertical visual angle of 1.8 degrees between the *up* and *down* stimulus locations. The task began with the presentation of four boxes, one after the other, over the course of 5,000 ms, with each box remaining onscreen for 25 % of the total 5,000 ms. The number of boxes increased in the same way as the digits in the memory task, although the length of time in which all the stimuli were presented remained the same. This meant that with each increase in level, the amount of time available to process each successive box decreased. Participants’ accuracy was calculated at the end of each level, and only those with an overall accuracy >80 % were permitted to continue on to the next level. The last level at which participants reached the 80 % accuracy level was recorded as their spatial processing span. Accuracy was calculated as the average of all responses for a block of trials. This 80 % criterion was set according to the standard cutoff reported in the TBRS literature (e.g., Barrouillet et al., 2007; Vergauwe et al., 2009, 2010). Performance below this level is deemed insufficient to disrupt concurrent memory storage due to unreliable cognitive load. Using this cutoff in our own experiments allows some comparison with previous research while providing a sensitive measure of verbal processing similar to our 4/5 memory span procedure.

The dual-task conditions combined the memory and processing tasks and were identical to the single-task memory span condition except that, following the presentation of the last memory item, there was a 1,000-ms fixation followed by the spatial processing task for 5,000 ms. Upon completion of the processing task, participants entered the previously presented numbers in the same way as in the single-task condition. Participants were instructed to perform both tasks as accurately as possible—neither task was labeled or implied to be the primary/secondary task. Participants completed four dual-task conditions, each with the spatial processing task set at different difficulty (hereafter referred to as *processing load*) levels based on each participant’s individually measured spatial processing span. For example, a participant who had reached a spatial processing span level with six boxes/5,000 ms would complete the four dual-task conditions with the processing task set at five (Span -1), six (Span =), seven (Span +1), and eight (Span +2) boxes/5,000 ms. The benefit of a titrated demand system, as opposed to having all participants complete the processing task at the same level, is that one can be confident that each participant is given *high* and *low* demand tasks based on their own measured ability. Dual-task memory spans were calculated in the same way as in the single-task condition.

### Results

#### A note on analyses

We report both *p* values and Bayes factors for each analysis. The use of frequentist statistics facilitates comparison with previous literature and are interpretable by a wide audience, whereas Bayes factors benefit from their ability to contrast two hypotheses that may or may not include a null hypothesis.

Bayes factors were calculated using the BayesFactor package (Rouder, Morey, Speckman, & Province 2012) in R (R Core Team, 2013). Priors for fixed effects were set at the default level of *medium* $\left(\sqrt[2]{2}/2\right)$. Null hypotheses refer to intercept-only models.

#### Memory span

**Fig. 1:**
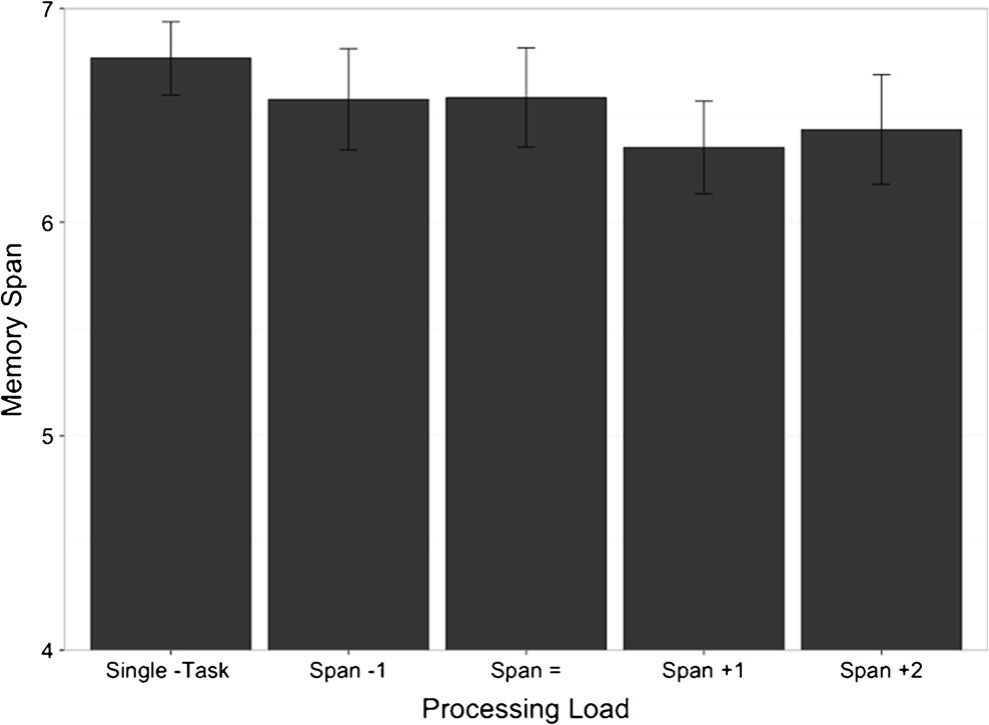
Experiment 1—Mean memory span scores (with standard errors) in single- and dual-task conditions

The effect of spatial processing on participants’ verbal memory performance was analyzed via a one-way repeated-measures analysis of variance (ANOVA). The analysis compared memory scores in the “Span -1,” “Span =,” “Span +1,” and “Span +2” conditions with participants’ single-task scores and revealed no significant main effect of processing load, *F*(4, 92) = 1.103, η_p_^2^ = .046, *p* = .360 (see Fig. 1).

Bayes factors larger than 1 indicate evidence for a model (in this case, an effect of processing task load), whereas Bayes factors smaller than 1 indicate evidence against a model (here, evidence *for* an *intercept only* or *null* model). The results of this analysis revealed around seven times more evidence in support of the null model compared to the model containing an effect of processing task load (BF = .14 ± .42 %).

#### Memory load × processing load analysis of processing accuracy

Mean single-task processing span was 7.58 (*SD* = 1.67). This meant that, on average, “Span -1,” “Span =,” “Span +1,” “Span +2” conditions were set at 6, 7, 8, and 9 items/5,000 ms, respectively. These levels translate to per-item presentation times of approximately 833 ms, 714 ms, 625 ms, and 556 ms, respectively.

**Fig. 2:**
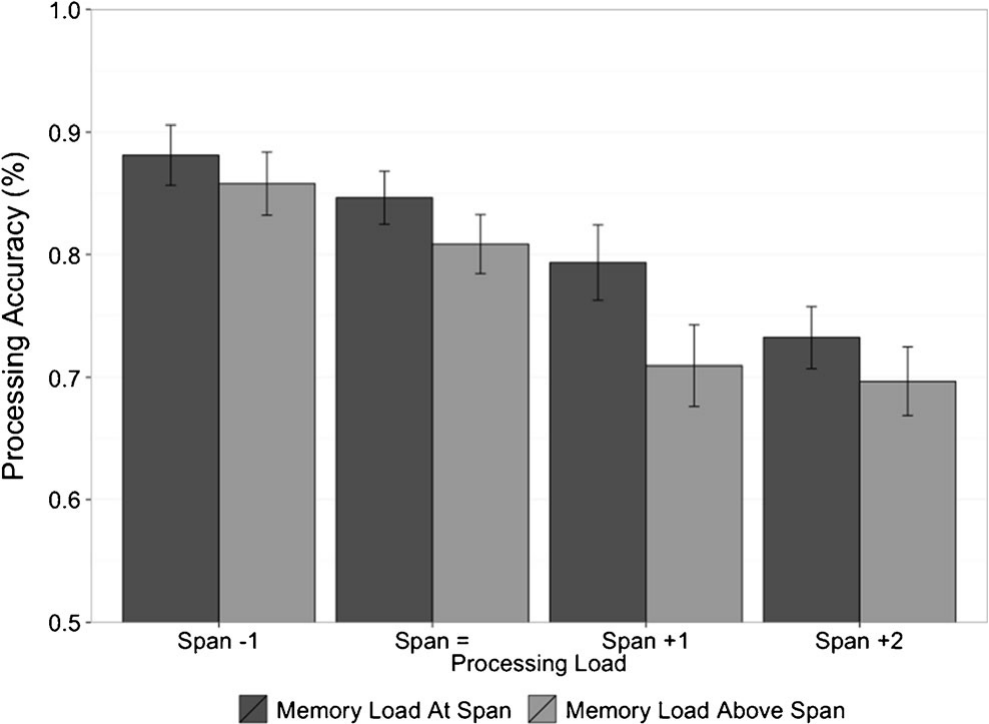
Experiment 1—Mean spatial processing accuracy (with standard errors) when memory load was at span and above span, shown across all dual-task conditions

Dual-task spatial processing accuracy was examined in relation to the level of memory load for each participant. Dual-task processing accuracy data were collected from the level at which participants failed the memory task criteria (memory load above span) as well as the level preceding this (memory load at span). This allowed a comparison of spatial processing accuracy in each dual-task condition when participant’s memory was at capacity and when their capacity was exceeded. According to Logie (2011), domain-general effects may be observed when working memory components are under heavy load that exceeds the capacity of a domain-specific system, and as such this analysis aimed to investigate whether spatial processing accuracy suffered as a result of the reallocation of resources to support above-span memory load. A 2 (memory load: at span vs. above span) × 4 (processing load: “Span -1” → “Span +2”) repeated-measures ANOVA revealed a significant main effect of both verbal memory load and processing load on spatial processing accuracy, *F*(1, 23) = 11.884, η_p_^2^ = .341, *p* = .002, and *F*(3, 69) = 18.096, η_p_^2^ = .440, *p* < .001, respectively (see Fig. 2). There was no significant interaction, *F*(1, 23) = 1.722, η_p_^2^ = .070, *p* = .171, and a Bayes factor analysis supported the main effects only model over the model containing an interaction by a factor of ~6 (BF = 6.36 ± .79 %).

The significant effect of memory load on processing accuracy reveals that accuracy in the concurrent spatial processing task was affected as memory load exceeded participants’ single-task span. This effect was not any larger when the processing load of the dual-task conditions increased further (as evident from the lack of interaction between memory load and processing load factors), suggesting that this trade-off is constant across dual-task conditions. If the effect increased with processing task demand this could suggest a single resource being diverted toward the more demanding memory task. Since this is not the case, this is evidence that memory performance is being supported by reallocation of resources rather than depending solely on a shared resource between processing and storage.

## Discussion

Experiment 1 provides evidence for a verbal memory store and rehearsal mechanism, which operate independently of concurrent spatial processing because participants’ verbal memory span was not significantly affected by the introduction of the secondary spatial processing task or with each subsequent increase in processing load between the “Span -1” and “Span +2” conditions. Although Fig. 1 appears to suggest subtle decreases in memory performance, the overall analysis demonstrates that these differences are not reliable: The effect size is small, and the Bayesian analysis revealed evidence for the null model. These results are consistent with temporary verbal memory relying on a separate resource, possibly the phonological loop, from online spatial processing rather than a shared resource.

In contrast to the verbal memory data, spatial processing accuracy was affected by the demand of the memory task: Participants’ processing accuracy was lower when participants’ memory was “pushed” above span. This supports Logie’s (2011) hypothesis regarding recruitment of domain-general resources when working memory components are under a load that exceeds their capacity. Figure 2 demonstrates this process effectively: As participants exceed their individual verbal capacity (i.e., task demand exceeded the capacity of their phonological loop), they recruit resources previously allocated to the spatial processing subtask to support their memory performance. The effect of memory load on spatial processing accuracy can therefore be interpreted as the effect of the reallocation of a processing resource once the phonological loop’s capacity is exceeded. It could, for example, reflect attempts to use a visuospatial strategy to retain some of the memory items in support of an overloaded phonological loop. A number of studies have shown the use of visuospatial codes and other codes (e.g., semantic) to retain sequences of verbal material (e.g., Borst et al., 2012; Logie et al, 1996; Logie, Della Sala, Wynn, & Baddeley, 2000; Logie et al., 2015; Saito et al., 2008). It is possible that recoding verbal memory items in a visual or other code is an attentionally demanding process that is susceptible to disruption when attention is required for some ongoing processing task.

As would be expected on any model, processing performance was affected by the increase in processing load, dropping below 80 % accuracy in the “Span +1” and “Span +2” conditions. As previously discussed, the TBRS literature reports that maintaining a level of processing performance above 80 % is essential to ensure that participants are indeed attending to the processing task. It is important to note that in our experiment single-task processing performance was measured as the last level at which participants performed the task with 80 % accuracy. The linear effect of increasing processing load between “Span -1” and “Span +2” is therefore expected due to the increasing difficulty of the task, and the drop in processing accuracy between “Span -1” and “Span =” appears no different than in other conditions despite the memory performance in these two conditions being near identical (see Fig. 1). If participants were indeed “giving up” on the processing task, we would expect to see performance drop to chance or below-chance levels, which could then account for the lack of cognitive load effect. Instead dual-task processing performance remains well above chance, mean performance doesn’t drop below 70 %, and >80 % accuracy is maintained in the “Span =” dual-task condition.

Within the context of a shared-resource approach to working memory, the aforementioned effect could be viewed as evidence of a general purpose resource facilitating both memory and processing tasks, with the primary memory task taking precedence over the secondary processing task. However, due to a lack of an interaction between memory load and processing load, this seems unlikely. If both memory and processing were relying equally on a single shared resource, then increasing the demand of the processing task should result in a progressively greater effect of memory load in each subsequent dual-task condition. Experiment 2 investigates whether this effect is limited to when memory load is set above span (evidence for separable resources when memory load is set at span level or below), or whether processing accuracy is improved at below-span memory load levels (evidence for a constant shared-resource trade-off).

## Experiment 2

Experiment 1 manipulated the demand of the processing task, revealing a significant effect of memory load on concurrent processing accuracy. We theorized that this effect of memory load was due to participants diverting resources from constantly updating information in the spatial processing task in order to support an overloaded phonological loop. Memory load was not directly manipulated and instead relied on an analysis of processing accuracy at the level participants failed the memory component of the dual-task conditions and at the highest level at which they succeeded. Experiment 2 aimed to directly compare spatial processing performance with varying levels of concurrent verbal memory load.

### Method

Experiment 2 used the same materials and followed the same procedure as Experiment 1 except that in the dual-task conditions we remeasured spatial processing span with the memory task set at levels based around each participant’s single-task memory span (again labeled “Span -1,” “Span =” “Span +1,” and “Span +2”). Twenty-four participants took part in the experiment in exchange for course credit (four male and 20 female, mean age = 19.0 years, range = 18–29 years).

### Results

#### Spatial processing

The mean single-task processing span for participants was 8.13 (*SD* = 1.12). Spatial processing was analyzed by comparing the accuracy from the highest common span level of the single- and dual-task conditions. For example, if a participant had reached eight boxes/5,000 ms in the single-task condition, and six boxes, seven boxes, six boxes, and five boxes in each of the dual-task conditions, the accuracy scores (% correct) from each condition’s five-boxes level would be compared. Conducting the analysis in this way allowed us to maintain a methodology near identical to Experiment 1 yet permitted a more precise comparison of processing accuracy scores rather than maximum span level reached under each condition. The mean highest common span level was 5.92 (*SD* = 1.72).

**Fig. 3:**
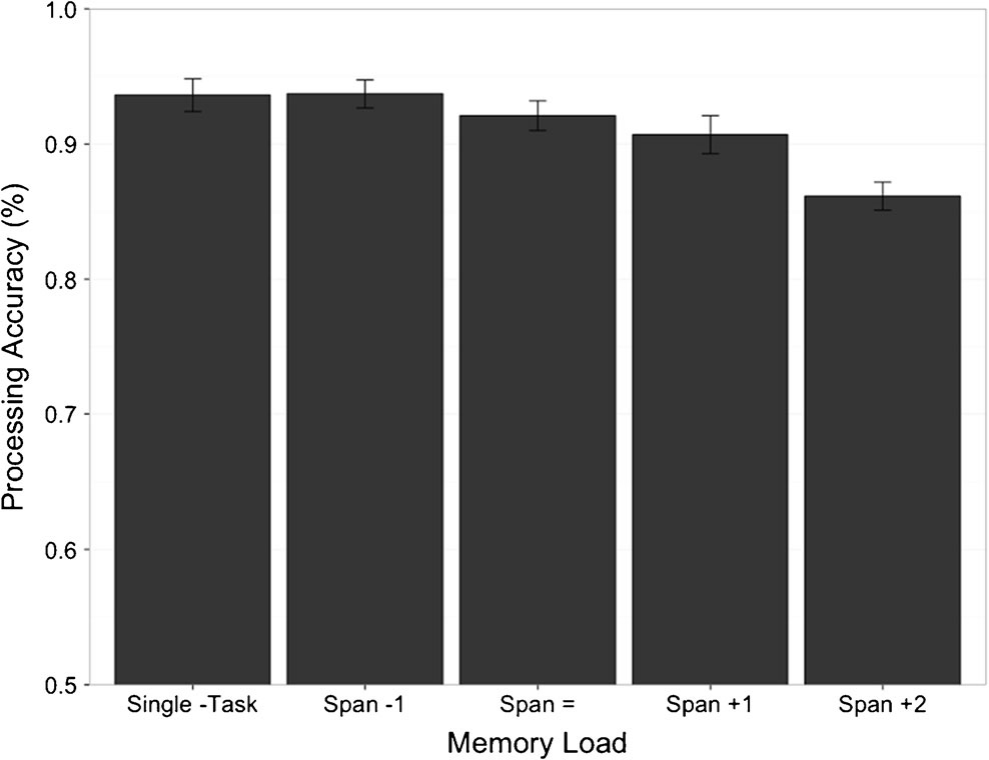
Experiment 2—Mean spatial processing accuracy (with standard errors) from single- and dual-task conditions

Scores were compared via a repeated-measures ANOVA and revealed a significant main effect of memory load on spatial processing accuracy, *F*(4, 92) = 8.325, η_p_^2^ = .266, *p* < .001 (see Fig. 3). Repeated-measures contrasts revealed no significant differences in accuracy between each successive condition (all *p*s > .243) except “Span+1” and “Span+2” conditions, *F*(1, 23) = 7.600, η_p_^2^ = .248, *p* = .011. These results reveal that as the memory load increased, performance on the concurrent spatial processing task decreased, with the largest effect appearing between the “Span+1” and “Span+2” conditions. Figure 3 strongly supports the conclusion that there is a lack of an effect of memory load between the single-task and “Span-1” conditions, in line with the hypothesis that the recruitment of additional resources only occurs once the verbal memory store’s capacity is exceeded.

We also analyzed reaction times to investigate whether we replicated Vergauwe et al.’s (2014) findings. It is important to note that Vergauwe et al.’s processing task was participant paced, whereas our task was experiment paced, and so we had to take steps to avoid artifacts in our data due to nonresponses. In calculating participants’ mean reaction time for each memory load condition, we removed any nonresponse trials as well as trials in which reaction time was <250 ms. An analysis of these data revealed no effect of memory load on reaction time: *F*(.206), ηp^2^ = .012, *p* = .789. A Bayesian analysis found ~12 times more evidence for the null model (BF = .08 ± .45 %).

#### Processing load × memory load analysis of memory accuracy

**Fig. 4:**
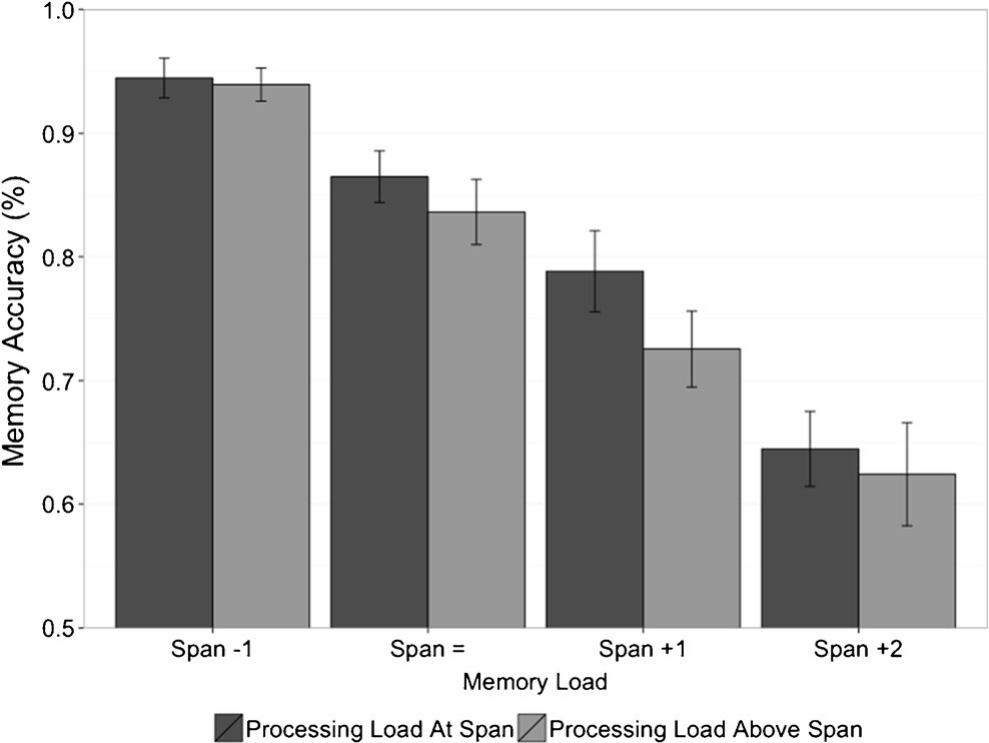
Experiment 2—Mean memory accuracy (with standard errors) when processing load was at span and above span, shown across all dual-task memory-load conditions

Mean single-task memory span was 6.18 (*SD* = .86). We compared performance in trials for the most demanding level of each dual-task condition with the lower load trials from the preceding block. Because spatial processing performance was the critical factor in passing each span level, these analyses aimed to compare concurrent memory storage (measured as a percentage of total memory items recalled in a block of trials) when participants’ spatial processing was operating at span and when it was operating above span. In Experiment 1 the analysis indicated that attentional resources were being diverted from the spatial task in order to support memory performance. A 2 (processing load: at span vs. above span) × 4 (memory load: “Span -1”→ “Span +2”) repeated-measures ANOVA revealed a significant effect of memory load on memory accuracy, *F*(3, 69) = 56.362, η_p_^2^ = .710, *p* < .001, and a significant effect of processing load, *F*(1, 23) = 5.087, η_p_^2^ = .181, *p* = .034, but no significant interaction, *F*(3, 69) = .618, η_p_^2^ = .026, *p* = .606 (see Fig. 4). A Bayesian analysis revealed that there was around nine times more evidence for main effects only compared to main effects with an interaction (BF = 9.54 ± .45 %). However, the Bayesian analysis also revealed little evidence for an effect of processing task load on memory performance, with the model containing *memory load only* being supported by a Bayes factor of 1.37 ± .71 % over the *memory load + processing load* model.

It is important to note that although participants’ memory accuracy decreases with increasing memory loads, performance is still high (remaining above 70 % in the “Span +1” condition). Relating this back to our hypothesis that storage draws upon additional resources once memory capacity is exceeded, the fact that memory performance does not drop to very low or chance levels in Fig. 4 supports the interpretation that participants are recruiting the similar resources as those responsible for the processing task (resulting in the drop in processing accuracy seen in Fig. 3) which enables them to maintain memory performance under demanding dual-task conditions.

### Discussion

Experiment 2 was successful in demonstrating the effect of diverting resources away from spatial processing to support increasing memory demands (as was observed in the Memory Load × Processing Load analysis in Experiment 1). As the demand of the memory task increased, participants appeared to rely more on resources also involved in the online processing of spatial stimuli, (although the only significant difference was between the , conditions).

Our reaction time analysis did not replicate Vergauwe et al.’s (2014) findings; however, this is likely due to both the aforementioned differences between our procedures and the fact that our participants were not under any articulatory suppression (Vergauwe et al.’s largest effects where in the articulatory suppression conditions, with smaller effects in the nonsuppression condition). It is possible that Vergauwe et al.’s participants were intentionally answering more slowly when under high memory load in an attempt to maintain performance through attentional refreshing or the use of some other mechanism, resulting in larger changes in reaction times than possible under our procedure.

The memory accuracy ANOVA revealed a small effect of processing task load on memory performance, whereas the Bayesian analysis provided very weak (or *anecdotal*; Wetzels & Wagenmakers, 2012) evidence *against* an effect of processing load. Therefore, if there is any processing load effect on memory accuracy (see Fig. 4) it is likely smaller than the effect of memory load on processing accuracy (see Fig. 2). Combined with the different patterns of data in Figs. 1 and 3, this supports our hypothesis that the processes involved in the spatial task supplement memory performance rather than both tasks relying equally on a single shared resource. It is possible that the small drop in memory performance results from the increased number of errors in the higher processing load condition, leading to a capture of attention due to error processing and therefore limiting the use of attention to support memory (see Lewandowsky & Oberauer, 2009, for an investigation of the possible effect of post-error slowing on memory performance).

## General discussion

We aimed to investigate the hypothesis that working memory comprises multiple components, each of which has its own capacity, and that when the capacity of a particular component is exceeded then performance can be supplemented by automatic or intentional recruitment of other resources, for example, through the use of visual codes for verbal material (e.g., Logie et al., 2000; Saito et al., 2008), the spontaneous use of some attention-demanding cognitive strategy other than subvocal rehearsal (e.g., Logie et al., 1996), attentional refreshing (e.g., Barrouillet et al., 2004, 2007), or the involvement of retrieval of memory traces from activated secondary or long-term memory (Logie, 1995; Unsworth & Engle, 2006, 2007).

Experiment 1 revealed no significant effect of increasing processing demand on concurrent retention and subsequent recall of memory items set at span for each participant. This provides further support (Baddeley, 1992; Camos et al., 2009; Logie, 1986) for a separation of temporary verbal memory storage from concurrent spatial processing. The analysis of processing accuracy revealed a significant drop in spatial processing performance once participants were “pushed” beyond their verbal memory capacity. We interpret this as an indication of a reallocation of attention from the processing task in support of the increased demand of the memory task: Additional resources are recruited to support performance as the capacity of the verbal store is exceeded.

This reallocation of attention is further supported in Experiment 2, where the significant main effect of memory load on processing performance was largest between the two highest memory load conditions (“Span +1” and “Span +2”). The fact that there appears to be no difference between the single-task condition and the “Span -1” dual-task condition in Fig. 3 supports the hypothesis that attention reallocation only occurs once the verbal memory store is at or above capacity and not before.

Note that participants were given no instruction as to the priority of the two tasks—that memory performance was unaffected by concurrent processing load while processing accuracy suffered with increasing list lengths is interesting because it suggests that participants intentionally or unintentionally sacrificed processing performance to maintain memory performance. We can only speculate as to whether the differences between our observations and those reported in the TBRS literature are due not only to our manipulation of load according to the span of each participant but also to some differences in the framed importance of tasks. For example, our tasks are “blocked” whereas TBRS methodology usually alternates presentation of memory items with processing items; we provide roughly equal “practice” on each task in the form of single-task conditions, whereas TBRS experiments often focus on extensive practice conditions for processing tasks to ensure performance exceeds 80 % in the dual-task condition. The manipulation of instructed priority or differential practice for one or other task would be interesting to explore in future studies.

Although this pattern of data could be interpreted as evidence for a single resource shared between memory and processing tasks, the lack of a convincing processing load effect on memory (see Figs. 1 and 4) suggests that this is not the case. Memory performance does not appear to be affected by increased processing load. We interpret this result by suggesting that the domain-specific verbal memory store cannot aid in the processing of visual stimuli.

Another interpretation could be that memory always requires attention, and that it is only once tasks become very demanding that the effect is observed. However, the clear lack of effect between the single-task condition and “Span -1” dual-task condition in Fig. 4 would suggest that this is not the case, as would the relatively small drops between the “Span -1” and “Span =“ conditions. Along with the lack of interaction in the analysis of processing in Experiment 1 (see Fig. 2), and the relatively small drop in performance between single- and dual-task conditions for both memory and processing (see Figs. 1 and 3), these data suggest a switch of attention in support of above-span memory performance rather than an ongoing trade-off between the two tasks. It is also possible that single-task span procedures such as those used in the experiments reported here are not “pure” measures of verbal memory capacity: Participants may use some or all of the rehearsal/maintenance methods that are available to them (e.g., subvocal rehearsal, visual recoding, refreshing). Whether it is possible to isolate and measure a specialized verbal memory store operating without the support of other cognitive mechanisms is an appropriate next step for identifying specialized components in the working memory system.

The existence of a flexible working memory system of discrete components that function as and when required in concert to support the overall level of observed performance can account both for patterns of data that support separable processing and storage, and can account for results that point to a single, flexible general purpose shared attentional resource. A participant’s preferences for recruitment of additional resources to support verbal memory performance could partly explain individual differences in verbal working memory along with the baseline capacity of components. Dual-task costs (e.g., Logie & Duff, 2007) could also be explained by the involvement of attention in single-task conditions (“boosting” performance beyond what is capable by the phonological loop alone) or indeed the involvement of other modality specific components such as a visual cache (Logie, 1995) or temporary activation in long-term memory (Unsworth & Engle, 2007). When attention is then occupied by the secondary processing task during dual-tasking, or when other modality-specific components are occupied with concurrent tasks, this would result in memory performance dropping to the residual capacity provided by a specialized verbal memory store. The asymmetry between verbal and visual short-term memory in terms of susceptibility to interference (Morey, Morey, van der Reijden, & Holweg, 2013) could also be accounted for by attention-based “boosting” of single-task capacity. If visual memory capacity is smaller than verbal temporary memory, or the procedures for maintenance are less practiced, then participants may resort to recruitment of additional resources at lower visual memory loads than for verbal loads, resulting in a more pronounced dual-task cost for the former. We have some preliminary data supporting this hypothesis from an experiment investigating the effects of concurrent spatial processing on visual memory span (Doherty & Logie, 2013).

Scientific debates can and do aid the progress of science but can also self-perpetuate and waste scientific energy (e.g., Newell, 1973), particularly if opponents in the debate maintain entrenched positions or focus on evidence that supports rather than challenges their views. We hope the data we present here go some way to resolving the multiple-component versus shared resource debate, which we believe has been perpetuated by a focus on different research questions by different research groups (i.e., overall capacity limits vs. the underlying structure of working memory). The investigation of different research questions has led to opposing models that may be more compatible than they might appear. Recent research conducted by the TBRS group has focused on the role of the phonological loop within a shared-resource working memory system (e.g., Camos & Barrouillet, 2014; Mora & Camos, 2013, 2015), and the experiments reported here, based on titrating cognitive load for each participant, indicate how, and under what circumstances, a domain-general resource within a multiple component system may support overall task performance. Future research could focus on explaining the contrasting core phenomena in the working memory literature by assuming a flexible working memory system comprising separable domain-specific and domain-general components all contributing to an emergent general working memory capacity.
